# Studying the effects of dietary body weight-adjusted acute tryptophan depletion on punishment-related behavioral inhibition

**DOI:** 10.3402/fnr.v59.28443

**Published:** 2015-08-11

**Authors:** Tilman J. Gaber, Vita L. S. Dingerkus, Molly J. Crockett, Sarah Bubenzer-Busch, Katrin Helmbold, Cristina L. Sánchez, Brigitte Dahmen, Beate Herpertz-Dahlmann, Florian D. Zepf

**Affiliations:** 1 Clinic for Child and Adolescent Psychiatry, Psychosomatics and Psychotherapy, RWTH Aachen University, Aachen, Germany; 2 JARA Translational Brain Medicine, Aachen & Jülich, Germany; 3 Department of Experimental Psychology, University of Oxford, Oxford, United Kingdom; 4 Institute for Neuroscience and Medicine, Jülich Research Centre, Jülich, Germany; 5 Department of Child and Adolescent Psychiatry, School of Psychiatry and Clinical Neurosciences & School of Paediatrics and Child Health, Faculty of Medicine, Dentistry and Health Sciences, The University of Western Australia, Perth, Australia; 6 Specialised Child and Adolescent Mental Health Services (CAMHS), Department of Health in Western Australia, Perth, WA, Australia

**Keywords:** acute tryptophan depletion, serotonin, executive functions, decision-making, gender

## Abstract

**Background:**

Alterations in serotonergic (5-HT) neurotransmission are thought to play a decisive role in affective disorders and impulse control.

**Objective:**

This study aims to reproduce and extend previous findings on the effects of acute tryptophan depletion (ATD) and subsequently diminished central 5-HT synthesis in a reinforced categorization task using a refined body weight–adjusted depletion protocol.

**Design:**

Twenty-four young healthy adults (12 females, mean age [SD]=25.3 [2.1] years) were subjected to a double-blind within-subject crossover design. Each subject was administered both an ATD challenge and a balanced amino acid load (BAL) in two separate sessions in randomized order. Punishment-related behavioral inhibition was assessed using a forced choice go/no-go task that incorporated a variable payoff schedule.

**Results:**

Administration of ATD resulted in significant reductions in TRP measured in peripheral blood samples, indicating reductions of TRP influx across the blood–brain barrier and related brain 5-HT synthesis. Overall accuracy and response time performance were improved after ATD administration. The ability to adjust behavioral responses to aversive outcome magnitudes and behavioral adjustments following error contingent punishment remained intact after decreased brain 5-HT synthesis. A previously observed dissociation effect of ATD on punishment-induced inhibition was not observed.

**Conclusions:**

Our results suggest that neurodietary challenges with ATD Moja–De have no detrimental effects on task performance and punishment-related inhibition in healthy adults.

The neurotransmitter serotonin (5-HT) is known to play a key role in many aspects of the human neurobehavioral system. Decreased availability of brain 5-HT is thought to be associated with a number of psychiatric conditions and pathological behaviors, such as depressive disorders, impulsivity and risk-taking behavior, alcohol abuse, anxiety disorders, other difficulties in emotional and regulatory functioning (1), eating disorders (2), and attention disorders (3). Specifically, 5-HT is involved in controlling motor output and behavioral inhibition (4) and the processing of aversive outcomes (5).

To study the effects of central nervous system 5-HT function, several methods, such as assessments of the 5-HT metabolite 5-Hydroxyindoleacetic acid (5-HIAA) in cerebral spinal fluids, the prolactin fenfluramine challenge test (6), and dietary approaches such as acute tryptophan depletion (ATD) (7) have been used. Briefly, the underlying principles of the dietary ATD procedure involve the oral administration of an amino acid (AA) beverage that lacks the essential amino acid tryptophan (TRP), the physiological precursor of 5-HT, which leads to a diminished rate of synthesis of 5-HT in the central nervous system due to decreased substrate availability (8–11). A newly developed body weight–adapted ATD challenge procedure termed Moja-De (8, 9, 12) has shown to be an effective and safe dietary method to transiently reduce central nervous system 5-HT synthesis to investigate the effects of lowered central nervous 5-HT availability on different aspects of human behavior (13, 14) including cognitive (12, 15–17) and affective (18) processes in healthy adults (9, 16, 17) and patient populations (13–15, 18).

Studies investigating the effects of dietary ATD administration on cognition and behavior have produced diverse and mixed results (19). Brain 5-HT availability as altered by dietary ATD protocols has been linked to inhibitory and aversive functioning (4, 20). The joint presence and interplay of inhibitory demands and error-related punishment in common behavioral inhibition tasks impedes the disclosure of the specific role of brain 5-HT in aversive processing. The question remains unanswered, whether 5-HT modulates motor responses, sensitivities toward the prospects of punishment, or behavioral adjustments following aversive outcomes. Recently, a novel task designed to selectively assess the effects of ATD on inhibition and aversive responding was implemented in healthy adult subjects (21), and the results suggested that dietary ATD administration and decreased brain 5-HT synthesis lead to significant reductions in punishment-induced inhibition, while leaving general motor inhibition and global sensitivity to aversive outcomes unaffected. The authors of this study concluded that 5-HT might be critical for neither punishment processing nor behavioral inhibition but might be specifically critical for the inhibition that is induced by the prospect of aversive consequences (21). In light of these findings, we sought to apply a more refined dietary ATD procedure to this novel behavioral inhibition task.

The body weight–adapted Moja-De ATD protocol has shown to have significantly fewer side effects (nausea, headaches, etc.), which allow its use in children and adolescents (14, 22, 23). Moreover, this particular dietary ATD protocol was recently validated in an animal model and shown to specifically reduce brain 5-HT synthesis in two strains of mice (8).

Here, we conducted a reinforced categorization task originally introduced by Crockett et al. (21) using a further developed, body weight–adapted dietary ATD protocol. Moja-De ATD has proven to be a highly effective method to temporarily lower serum and cortical TRP availability in rodents (8) and humans (9, 16) and resulted in fewer aversive side effects allowing its use in children and adolescents (14). Moreover, regarding the punishment-related processing of information, we modified the task conditions with respect to the performance consequences. The present study examined the effects of dietary ATD Moja-De administration on behavioral inhibition with an established task (21) that emphasizes intrinsic motivation without providing immediate and explicit performance-based monetary rewards. Moreover, gender confounds in ATD-related research have been reported (24), and gender-dependent differences in brain 5-HT synthesis have also been published (25). Therefore, we opted to balance gender in the present sample. As a result of our modifications to the protocol, we expected a lower rate of unintended aversive side effects associated with an optimized ATD-related dosing scheme and a higher global task adherence due to higher intrinsic motivation. Hence, we hypothesized a less pronounced detrimental effect of dietary ATD administration on response accuracy and response times (RTs) in the reinforced categorization task compared with previous findings.

## Methods

### Subjects

Twenty-four healthy adult volunteers (12 females/12 males; mean age=25.4 years) attended two test sessions on two different study days (Table 1 provides the characteristics of the analyzed study sample). The study protocol was approved by the local ethics committee and was in accordance with the Declaration of Helsinki. All participants provided oral and written informed consent and were financially compensated at the end of the second study day. The inclusion criteria were good mental and physical health and the absence of a personal history of developmental disorders, schizophrenia, affective disorders, psycho-organic syndromes, medication or drug use, organic diseases, pregnancy and low IQ (<85). Prior to the first session, the participants were screened for physical and psychiatric disorders via an interview that utilized a standardized diagnostic instrument (the SKIDPIT light; 26).

**Table 1:** Demographic data of the study sample (*n*=24)

Parameter	Total (*n*=24)	Males (*n*=12)	Females (*n*=12)	*p*
Age (years)	25.34 (2.09)	25.34 (2.43)	25.30 (1.69)	0.917 (ns)
Weight (kg)	70.54 (11.86)	80.08 (6.30)	61.00 (7.70)	<0.001 (***)
BMI (kg/m^2^)	23.04 (1.86)	24.01 (1.55)	22.07 (1.63)	0.007 (**)

The data are presented as the means and the standard deviations. Between-group comparisons (p) were conducted using independent two-tailed *t*-tests.

### Study design

To temporarily reduce brain 5-HT availability, we applied a body weight–adjusted ATD procedure in a randomized double-blind, within-subject, counterbalanced, repeated-measures crossover design. Participants’ morning arrivals at the study site were preceded by a 12-h overnight protein fast, and a standardized breakfast that was low in TRP. A part of the sample received ATD on day 1, followed by administration of a balanced control mixture containing TRP (BAL) on their second day of testing. The remaining subjects received the same challenge procedures in the reverse order. Testing days were spaced at least 7 days apart. Prior to the intake of the AA mixtures in an aqueous suspension, urine drug screenings, pregnancy tests for the female participants, and the collection of baseline blood samples (T0) were performed. In addition, three blood samples were taken 90 min (T1), 180 min (T2), and 270 min (T3) after AA challenge intake (ATD/BAL). Plasma levels of total TRP, free TRP, and the ratio of free TRP to other large neutral amino acids (LNAA) were assessed at all four time points. A more detailed description of the depletion protocol and an extensive laboratory assessment are the subject of different publications (9–11).

### Depletion procedure

The AA mixture was identical to that of the previously used Moja-De protocol (8, 9, 23) and contained the following AAs (dosage per 10 kg of body weight): l-phenylalanine (PHE 1.32 g), l-leucine (LEU 1.32 g), l-isoleucine (ILE 0.84 g), l-methionine (MET 0.5 g), l-valine (VAL 0.96 g), l-threonine (THR 0.6 g), and l-lysine (LYS 0.96 g). The BAL mixture contained the same quantities with an additional 0.7 g of TRP per 10 kg body weight. Nine participants (six females) received ATD on their first day of participation, and 15 participants (six females) received ATD on their second day of participation.

### Behavioral experiment

**Fig. 1:**
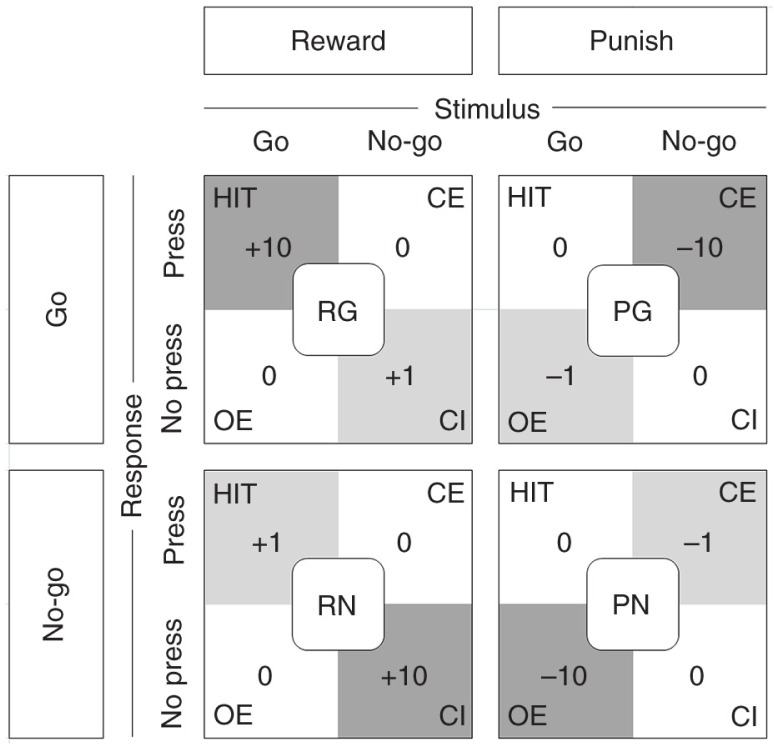
The Response-outcome contingency conditions consisted of four blocks of trials, each of which selectively rewarded correct responses or punished incorrect responses. Reward conditions emphasized correct go responses (reward go – RG) or correct response inhibition (reward no-go – RN). Punishment conditions emphasized consequences of incorrect go responses (punish go – PG) or incorrect response inhibitions (punish no-go – PN). Each block was associated with specific gains or deductions for correct or incorrect responding. For trials of the stimulus–response condition RG, correct Go responses (Hit) were associated with high gains (+10 points) and correct inhibitions (CI) were rewarded with moderate gains (+1 point), whereas all incorrect responding, that is, commission errors (i.e. pressing the button when not required, CE) and omission errors (i.e. not pressing the button when required, OE) had no consequences (±0). The same was true for the RN condition, except that the magnitude of gains for Hit and CI were swapped. During trials of the punishment conditions (PG and PN), incorrect responses (i.e. CE and OE) were accordingly associated with high (−10 points) and moderate (−1 point) deductions. Correct responses had no consequences (±0). Neutral blocks without gains or deductions were presented before and between the four reinforcement blocks. Subjects received feedback about their response outcomes immediately after each trial.

A specifically adapted, reinforced forced choice categorization task (go/no-go) was used to collect individual measures of executive inhibition, punishment-induced inhibition, and sensitivity to aversive outcomes. A detailed description of the task has been published by Crockett et al. (21). Briefly, the stimulus material consisted of bicolor checkerboards with a total of 25 rectangles. Depending on the task, these checkerboards contained either a greater or fewer number of the target color squares. Target to non-target square ratios were 16 to 9 or 9 to 16 for easy problems, and 13 to 12 or 12 to 13 for difficult problems. If the target squares were in the majority, a button press (‘go’) was demanded, and if the target squares were in the minority, withholding of the motor response (‘no-go’) was required. Easy and difficult trials were equally and pseudorandomly distributed in all blocks. Response outcomes were presented immediately after each trial and depended on response accuracies and the specific response bias condition. To investigate subjects’ responses to rewards and punishments, four distinct response–feedback conditions were applied (see Fig. 1). In order to selectively bias subjects' responding, correct motor responses were specifically reinforced by greater gains (Reward go and Reward no-go) or incorrect motor responses were punished by greater deductions (Punish go and Punish no-go). Figure 1 depicts all four response-outcome contingencies and the magnitude of gains and deductions for all possible task–response combinations. To prevent carryover effects from previous response bias conditions, blocks of neutral non-reinforced trials were interposed between the reinforcement blocks. The stimulus presentation durations were set individually based on the individual performance during a practice round prior to the actual measurement. Commission error rate (CE) and RTs of correct responses were assessed as outcome measures. For detailed description of the task see Crockett et al. (21). Our experimental settings were designed to allow for a replication of the original study with the two differences discussed above; that is, the specific ATD procedure and the parameters of the performance-based rewards.

### Data analysis

Serotonin manipulation was evaluated by analyses of the blood samples that were taken at four time points (before intake, and 90, 180, and 270 min after intake). Laboratory assessments included the levels of total plasma tryptophan, free tryptophan, and the ratio of free tryptophan to other large amino acids (fTRP/ΣLNAA). Analyses of the behavioral data were conducted following the procedures of Crockett et al. (21) with some minor differences. Our general behavioral analysis included preprocessed data regarding CE rates, response biases, and normalized RTs from all four experimental reinforcement conditions. Repeated-measures ANOVAs (SPSS 19) including gender as a covariate and treatment and feedback type and response bias (Go/No-go) as within-subject factors were conducted. Individually adjusted z-scores (normalized against matched non-reinforced trials) for RTs were processed and included in all computations. Motor response inhibition was assessed by examining the CE rates (i.e. incorrect no-go responses) and RTs of correct go responses. Reward and punishment signaling was assessed by measuring response adjustments based on response-outcome contingencies. The influence of ATD on discrimination of the prospects of small and large gains and losses was tested. The tendency to favor more beneficial responses in accordance with the outcome bias was assessed with the natural log of β (or ln(β)) from signal detection theory (27).

Punishment-induced inhibition was assessed by comparing the RTs of correct go responses in the punishment conditions to those of the reward and neutral conditions. RT scores were normalized into z-scores by matching them against the mean RTs of the corresponding blocks of neutral trials. To assess the immediate behavioral impact of experienced punishment on task performance, we compared the z-transformed RTs of trials following punishment with those of trials following non-punishment. Subjects underwent behavioral testing after a rest period of 180 min (i.e. after T2).

## Results

### Dietary brain serotonin manipulation

**Fig. 2:**
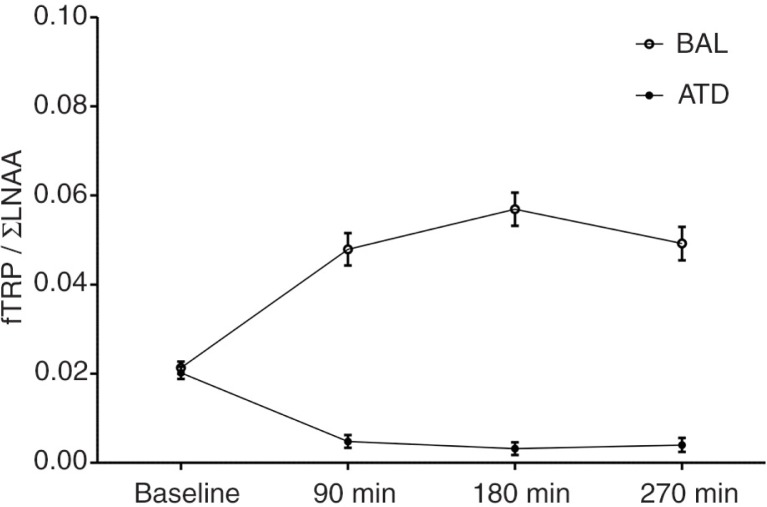
Plasma concentrations of free tryptophan relative to large neutral amino acids (fTRP/LNAA ratio) at baseline (prior to AA intake) and at three time points after the intake of the acute tryptophan depletion (ATD) or balanced amino acid load (BAL) mixtures. The behavioral tasks were performed 180 min after AA intake. The error bars depict the standard error of the mean (SEM).

Simple effects analysis revealed significant reductions in the plasma levels of free (fTRP; −28.0%; *t*_(23)_=3.350; *p*=0.003) and total TRP (tTRP; −29.2%; *t*_(23)_=3.139; *p*=0.005), and fTRP/ΣLNAA ratios (−84.1%; *t*_(23)_=13.528; *p*<0.001) at T2 (3 h after intake) for ATD relative to baseline. In contrast, TRP levels increased for all measures relative to baseline after BAL administration: tTRP (+581.4%; *t*_(23)_=21.345; *p*<0.001), fTRP (+1116.4%; *t*_(23)_=15.080; *p*<0.001), fTRP/ΣLNAA (+66.9%; *t*_(1,23)_=8.688; *p*<0.001; see Fig. 2). A gender-controlled, repeated-measures MANOVA revealed a highly significant two-way interaction between time point (baseline,+3 h) and dietary challenge (BAL/ATD): fTRP (*F*_(1,22)_=237.325; *p*<0.001), tTRP (*F*_(1,22)_=571.775; *p*<0.001), and fTRP/ΣLNAA ratio (*F*_(1,22)_=144.108; *p*<0.001). There was no significant between-subject effect of gender (fTRP: *F*_(1,22)_=1.677; *p*=0.209) and no interaction effects of gender and time point (fTRP: *F*_(1,22)_=1.498; *p*=0.234); gender and dietary challenge (fTRP: *F*_(1,22)_=1.638; *p*=0.214); or gender, time point, and dietary challenge (fTRP: *F*_(1,22)_=1.368; *p*=0.255). As reductions in plasma TRP levels indicate reduced TRP influxes into the brain, a subsequent reduction in substrate availability for brain 5-HT synthesis can be inferred (8, 9, 16, 28).

### Behavioral results

#### General effects of ATD and gender on task performance

Repeated-measures ANOVAs of error rates and mean RTs for valid trials were performed. They included the factors dietary challenge (BAL vs. ATD), task difficulty (easy vs. difficult), and the between-subject factor of gender (male vs. female). Administration of ATD resulted in significant differences in CE rates (*F*_(1,22)_=4.654; *p*=0.042) and RTs (*F*_(1,22)_=13.918; *p*=0.001) compared with BAL. Specifically, dietary ATD administration resulted in fewer errors (mean±SE; 16.2%±1.6%) and faster responses (503.09 ms±11.9 ms) compared with the balanced control condition (19%±1.6%; 559.28 ms±8.0 ms). Thus, impaired motor response inhibition due to ATD was not observed. Administration of ATD seemed rather to have facilitating effects on performance.

Mean RTs and CE rates did not vary significantly depending whether ATD was administered on day 1 or on day 2 but showed a trend for faster RTs on day 2 (*F*_(1,22)_=4.1; *p*=0.055). For a two-way interaction between treatment and treatment order, data showed a significant effect for RT (*F*_(1,22)_=12.2; *p*=0.002) but not for CE (*F*_(1,22)_=3.4; *p*=0.078).

Task difficulty (easy vs. difficult) strongly affected error rates and RTs. Due to the significant disparity of RTs across levels of difficulty (easy: mean=488 ms and difficult: mean=565 ms), these trials were not collapsed for analysis (*F*_(1,46)_=15.3; *p*<0.001), which, notably is a deviation from the procedures of Crockett et al. (21). Furthermore, the easy trials yielded very few CEs, which was in sharp contrast with the difficult trials (*F*_(1,46)_<128.9; *p*<0.001); thus, only the difficult trials were included in the analyses of the general effects of ATD on task performance that incorporated task difficulty. Further, from a methodological point of view, it seems inappropriate to collapse the response data across the difficulty levels. There is empirical evidence that suggests that easy and hard trials should be treated separately in discrimination tasks. In addition to others, Fleming et al. (29) reported that certain easy discriminative choices engage a neural basis that is distinct from that engaged by difficult choices. This differential engagement likely occurred in our paradigm because the easy trials may have been solved on a perceptive level, whereas the difficult trials potentially involved higher cognitive functions. In addition, a significant two-way interaction between task difficulty and dietary challenge was observed, and this interaction was due to more pronounced reductions in RTs for the difficult tasks under ATD compared with BAL (*F*_(1,22)_=6.681; *p*=0.017). No significant main (*F*_(1,22)_=1.655; *p*=0.212) or interaction effects were observed for the between-subject factor of gender.

#### ATD speeds behavioral responses

**Fig. 3:**
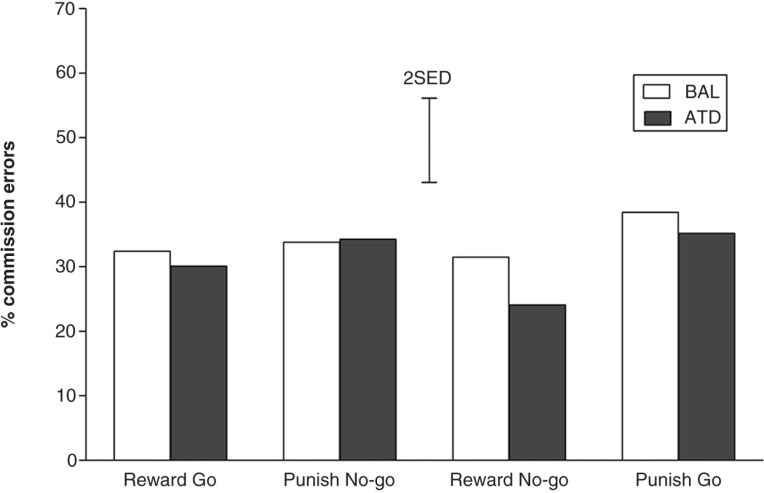
Rates of incorrect go responses (commission errors) for the difficult trials for all four experimental conditions under conditions of depleted or balanced tryptophan availability. The error bars depict the standard errors of the differences of the means (SED).

For the difficult trials only, the CE rates did not vary as a function of dietary challenge (*F*_(1,22)_=1.328; *p*=0.262) or response-outcome contingencies (*F*_(1,22)_=0.324; *p*=0.575). Participants committed an equally high proportion of CEs during the blocks that were biased toward go responses (reward go and punish no-go) and the blocks that were biased toward no-go (reward-no-go and punish-go). No interaction effects between response bias and dietary challenge (*F*_(1,22)_=1.348; *p*=0.258) or response bias and gender (*F*_(1,22)_=0.496; *p*=0.488) were detected (see Fig. 3).

**Fig. 4:**
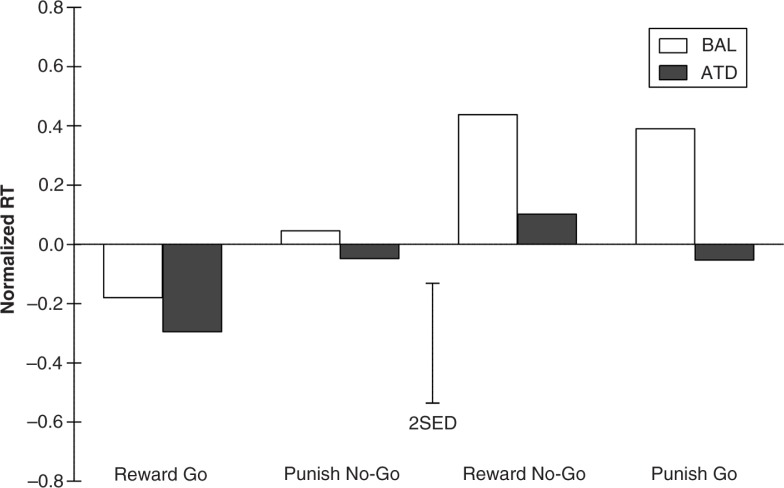
Normalized response times for all four experimental response-outcome contingency conditions under conditions of acute tryptophan depletion (ATD) and balanced amino acid load (BAL). The error bars depict the standard errors of the differences of the means (SED).

The analysis of RTs for correct go trials is presumably a more sensitive measure of motor response inhibition. RTs varied significantly as a function of response bias (*F*_(1,22)_=9.569; *p*=0.005) and dietary challenge (* F*_(1,22)_=14.628; *p*=0.001). As expected, responses were faster for trials biased toward the go response than trials biased toward the no-go response (see Fig. 4). Administration of ATD resulted in faster responses (or reduced slowing), which was also observed in the initial unbiased neutral block (−7.1%; *t*_(1,23)_=2.359; *p*=0.027). The test for a modulating effect of ATD on response bias (treatment x bias interaction) did not reach significance (*F*_(1,22)_=4.036; *p*=0.057). Thus, the expected impairment in motor response inhibition due to the administration of ATD was not observed. This finding is indicative of intact behavioral responding after ATD Moja-De. Again, gender had no general effect on CE rates (*F*_(1,22)_=0.545; *p*=0.468) or RTs (*F*_(1,22)_=0.374; *p*=0.547).

#### Punishment anticipation has no effect on behavioral inhibition

**Fig. 5:**
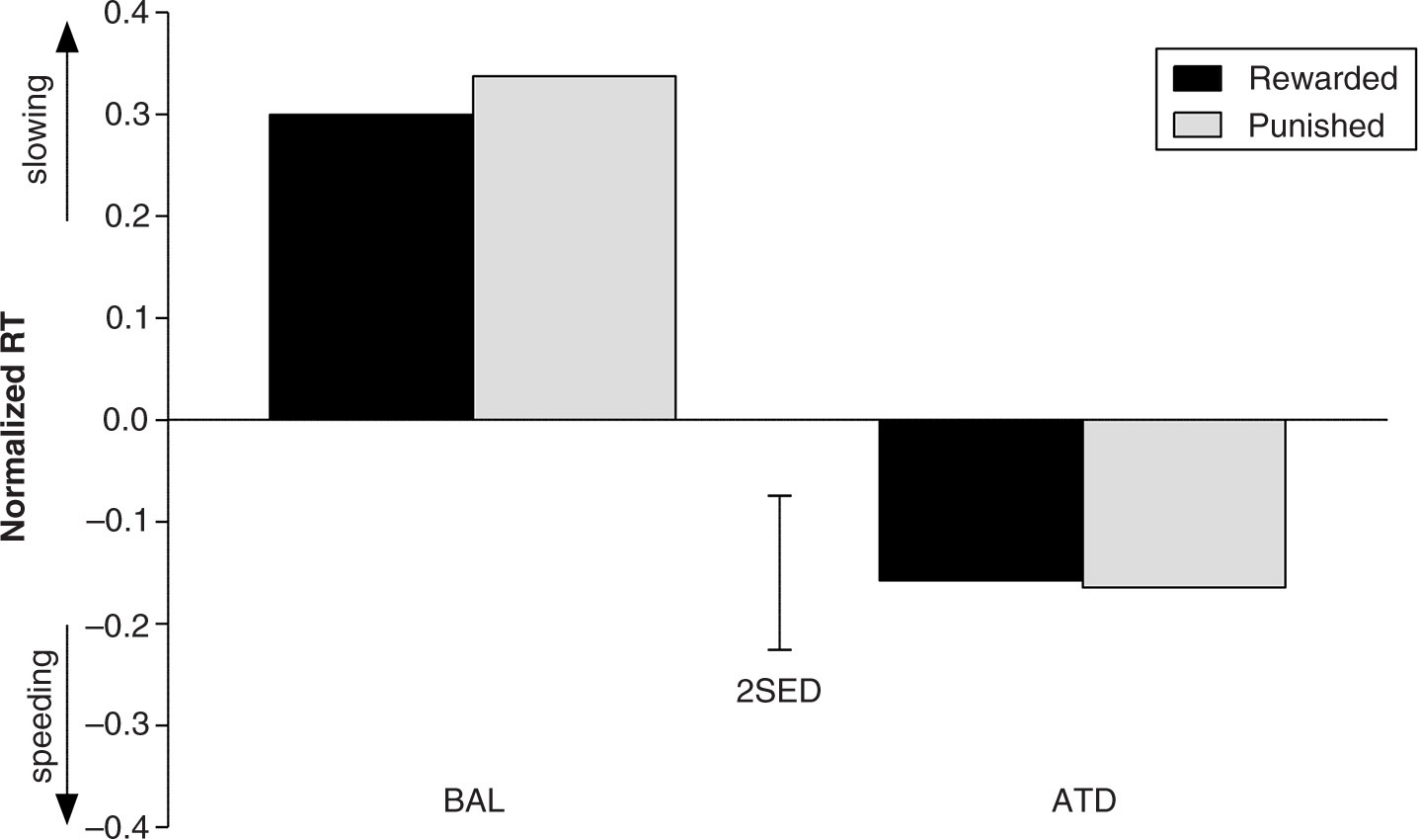
Effects of dietary challenge on response times in the reward and punishment conditions. No interaction effect of the reward and dietary challenge conditions was observed; thus, no effect of ATD on punishment-induced inhibition was observed. RTs were normalized against the neutral baseline. The error bars depict the SED.

The previously reported effects of punishment-induced behavioral inhibition after ATD administration (i.e. slower RTs in the punishment condition compared with the reward condition) did not replicate in our data (Fig. 5). The prospect of punishment for erroneous responding did not produce behavioral alterations in RTs (*F*_(1,22)_=0.002; *p*=0.969) in either the BAL (*t*_(1,23)_=−0.306; *p*=0.762) or ATD (*t*_(1,23)_=0.082; *p*=0.935) conditions. There was also no modulatory effect of dietary challenge administration on bias (*F*_(1,22)_=0.273; *p*=0.607).

#### Sensitivity to aversive outcomes is unaffected by reduced brain 5-HT synthesis

**Fig. 6:**
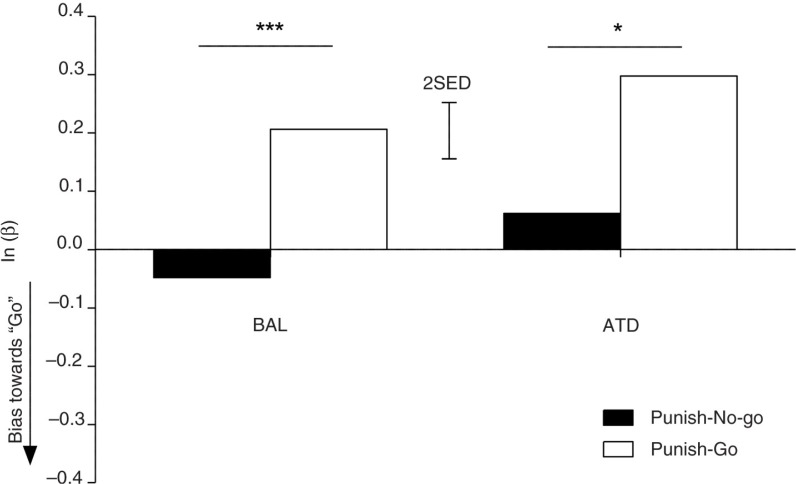
Punishment sensitivity. Comparison of the response biases in the punish-go and punish-no-go conditions. Negative values indicate biases toward go. The error bars depict the SED. *p<0.05; ***p<0.001.

To test the role of brain 5-HT availability in the prediction of aversive outcomes, we conducted a repeated-measures ANOVA on the response bias data (natural log of β) for the punished conditions only. Erroneous go responses were more severely punished during the punish-go trials, while no-go responses were more severely punished during punish-no-go trials. Therefore, intact signaling of aversive outcomes should be reflected by the tendencies to shift toward the go response in the punish-no-go condition and vice versa. The expected main effect of punishment bias was observed (*F*_(1,23)_=22.275; *p*<0.001). However, there was no modulatory effect of dietary challenge on punishment bias (*F*_(1,23)_=0.024; *p*=0.879). Thus, ATD administration did not alter sensitivities to aversive outcomes (see Fig. 6). During the ATD sessions, subjects were equally likely to favor the less punished response option as in the BAL session (*F*_(1,23)_=2.636; *p*=0.118).

#### Linear regression of the degree of ATD and behavioral slowing

To assess whether the degree of reduction in fTRP levels altered punishment-induced inhibition, a linear regression analysis was performed with the differences in the normalized RTs between the reward and punishment conditions as the dependent variable and the changes in fTRP/ΣLNAA as the predictor. No significant effect of relative fTRP availability on RT differences between the reward conditions was observed (*r*=0.234; *t*_(23)_=1.128; *p*=0.272).

#### Behavioral impact of punishment on subsequent performance

In addition to the analysis of the effects of anticipated punishment, we analyzed behavioral responses immediately after received punishment. In accordance with Crockett et al. (21), RTs after punishment and non-punishment trials were compared in terms of dietary challenge with gender included as a covariate. We observed significant main effects for the factors dietary challenge (*F*_(1,23)_=7.319; *p*=0.013) and prior punishment (*F*_(1,23)_=16.694; *p*<0.001). The subjects’ responding on trials that immediately followed punishment was significantly slower (mean±SE; 0.493±0.106) compared with trials after non-punishment (mean±SE; 0.016±0.049). This effect was significant under both challenge conditions, ATD (*t*_(23)_=−5.615; *p*<0.001) and BAL (*t*_(23)_=−3.644; *p*=0.001). However, there was no significant interaction effect between dietary challenge and prior punishment (*F*_(1,23)_=1.224; *p*=0.281). In contrast to the hypothesized attenuating effect of ATD on sensitivity to punishment, the subjects’ ability to adjust their responding after punishment was not impaired.

## Discussion

The goal of the present study was to investigate whether the previously reported diminished punishment-induced behavioral inhibition due to the influence of reduced central nervous 5-HT synthesis would extent to a body weight–adapted dietary ATD protocol. Our study detected marked reductions in absolute and relative plasma TRP levels after ATD administration. There is converging evidence that ATD Moja-De decreases brain 5-HT synthesis in rodents (8) and humans (9). Moreover, ATD-Moja-De was used in combination with neuroimaging techniques (30), which is of particular importance when it comes to studying the impact of AD related precursor availability and its impact on brain function (31). However, the concept of dietary ATD administration has recently been questioned with respect to the specificity of its effects on central nervous system 5-HT (32), but there is clear and convincing evidence that ATD diminishes central nervous 5-HT synthesis (8, 9, 28, 33).

Regarding behavioral performance after AA intake, the previously reported ATD-related reductions in response accuracy did not replicate in this study. Our data support previous findings that ATD does not influence executive function performance per se (19). The observed performance improvements after ATD administration, particularly faster responding, have previously been reported for memory tasks (34–37), executive functions (38–40), and emotion recognition tasks (41). This raises the question of whether the previously reported performance alterations were due to reduced central nervous 5-HT synthesis itself or due to nonspecific accompanying effects. Presumably reduced side effects by the body weight–adapted ATD protocol could account for a reduction of biasing of behavioral performance. Thus, the minimization of aversive effects may have contributed to the minimization of detrimental effects on performance that have previously been observed after ATD. It has been suggested that ATD might affect the cognitive and executive performance of specific populations; that is, populations with schizophrenia (42), Parkinson's disease (37), remitted depressed patients (43), subjects with family histories of alcoholism (44), and menopausal women (45).

It has been argued that processing of reward and punishment accesses separate motivational systems and is also mediated by distinct underlying neural substrates. On the one hand, reward processes are thought to be associated with dopaminergic neurons in the dorsal and ventral striatum (46). On the other hand, punishment-related processes have been linked to activation predominantly in the inferior frontal gyrus and, specifically, in the insular cortex. From a neurochemical viewpoint, punishment is believed to be linked to the serotonergic system that originates in the median raphe nucleus (47).

Some methodological issues of the present findings also deserve consideration. Participation in the present study was monetarily compensated at the end of each session. However, in contrast to the original study design, task performance was not immediately reinforced by monetary rewards. Moreover, the better accuracy and speed performances of our sample compared with the sample of Crockett et al. (21) suggest that motivation was generally higher, more persistent, and that the experimental compliance was better in this study. Moreover, the sample of the original study displayed high CE rates, partially around chance level (21). This might indicate an excessive tradeoff toward (global) reward optimization at the expense of the compliance with the task instructions, triggered by the prospect of immediate monetary gains.

In contrast, social acceptance has been shown to serve as a strong reinforcer (48). It has been reported that social gratification processes rely on neural pathways that are comparable to those of monetary reward (49, 50). In addition, negative social reinforcements can be applied and can actually be perceived as punishments in experimental settings. In this context, aversive conditioning (loss/punishment) with primary (sounds/odors) (51) and secondary reinforcers (52) modulate perception and influence discriminatory sensitivity for loss-related stimuli. Consequently, altered perceptual thresholds and generalization effects have been observed (52). Because reward and motivational mechanisms occur at the perceptual level, task-related top–down processes may be compromised and thus result in inferior task performance despite elevated motivational levels. Loss of discriminatory sensitivity as a result of punishment conditioning could lead to reduced discriminatory accuracy (i.e. the commission of more punishable errors or decisions) and result in even more aversive outcomes. In other words, the consequences of erroneous decisions could directly lead to elevated rates of erroneous decisions in a discrimination task. Finally, the question of whether gains and losses differently impact the mechanisms relevant to perceptual decision-making has been raised, particularly with respect to the recent evidence of the distinct processing of loss and gain prospects in the brain (53, 54).

Notably, the characteristics of the samples varied between the two studies. Whereas the original study (21) included nearly twice as many female as male subjects, our aim was to ensure an equal gender distribution in the present study. Brain 5-HT synthesis rates have been found to be significantly lower in female subjects than in with males (25). A large meta-analysis reported that females are more vulnerable to the effects of dietary ATD administration in terms of cognitive processing (55). There is also evidence that gonadal steroids have regulatory influences on the reward system in female rats (56). While some studies have exclusively included male (53) or female subjects (57), we decided to include an equal number of both and include gender as a covariate in our statistical analysis. Several behavioral studies have shown that females exhibit greater memory impairments after ATD (19) than males. Administration of ATD has been shown impair declarative memory to a greater extent in females (55). In addition, the negative mood-inducing effects of dietary ATD have been reported to be greater in women than in men (58–60). Imaging studies suggest marked gender differences in social and monetary reward paradigms in terms of task performance on the behavioral and the neural levels. For example, male subjects are behaviorally more sensitive to increasing levels of monetary reward compared with social reward. In addition, males exhibit a more widespread pattern of brain activation in response to anticipated monetary (but not social) rewards than female subjects. In contrast, women exhibit a balanced pattern of cortical activation pattern in response to increasing levels of social and monetary rewards (61). Given these sex-related differences, future studies should carefully control for gonadal steroid levels (menstrual cycle), and imaging studies should incorporate designs that factorize sex hormone levels and activations of the neural substrates of the reward and punishment systems related to behavioral performance. However, our body weight–adapted dietary depletion protocol was not affected by gender confounds in this sample. Apart from the overall slower behavioral responding of females, no task or task-by-treatment interaction effects were observed.

Besides the above-mentioned advantages of the ATD Moja-De protocol, this study has some limitations. While the present study was carefully balanced in terms of gender and homogenous in terms of demographic parameters, the sample size of the present study was rather small but comparable with other previous experimental dietary challenge studies using ATD. However, there was no significant interaction between treatment, treatment order and gender. Also, it should be noted that menstrual cycle and hormone status of the female participants were not controlled for in this particular study as it was done in a subsequent study (16). However, although this is common practice in most ATD-related mixed gender studies, a possible modulatory effect of the order of challenge administration and hormone status on our female sample cannot be ruled out, and should be addressed in future studies.

## Conclusions

In summary, the present study indicates that the body weight–adapted dietary Moja-De ATD procedure is a useful physiological method for effectively reducing central nervous 5-HT synthesis in healthy adult volunteers. This method produced marked effects on behavioral performance in an elaborate selective inhibition response task. Reduced central nervous system 5-HT levels correlated with faster responding and enhanced overall performance accuracy (i.e. reduced error rates). In the light of the discrepancies between the present findings and previous findings, further explorations of the specific role of 5-HT in aversive prediction and the relation of 5-HT to the reward system should be conducted.
